# Supplementary material for: Interaction of two MADS-box genes leads to growth phenotype divergence of all-flesh type of tomatoes
Source: Nat Commun. 2021 Nov 25;12:6892. doi: 10.1038/s41467-021-27117-7 (PMC8616914; doi:10.1038/s41467-021-27117-7)
Supplement: Supplementary file 1 — Supplementary Information [file 41467_2021_27117_MOESM1_ESM.pdf]

Interaction of two MADS-box genes leads to growth phenotype divergence of all-flesh type of tomatoes

Huang *et al.*,

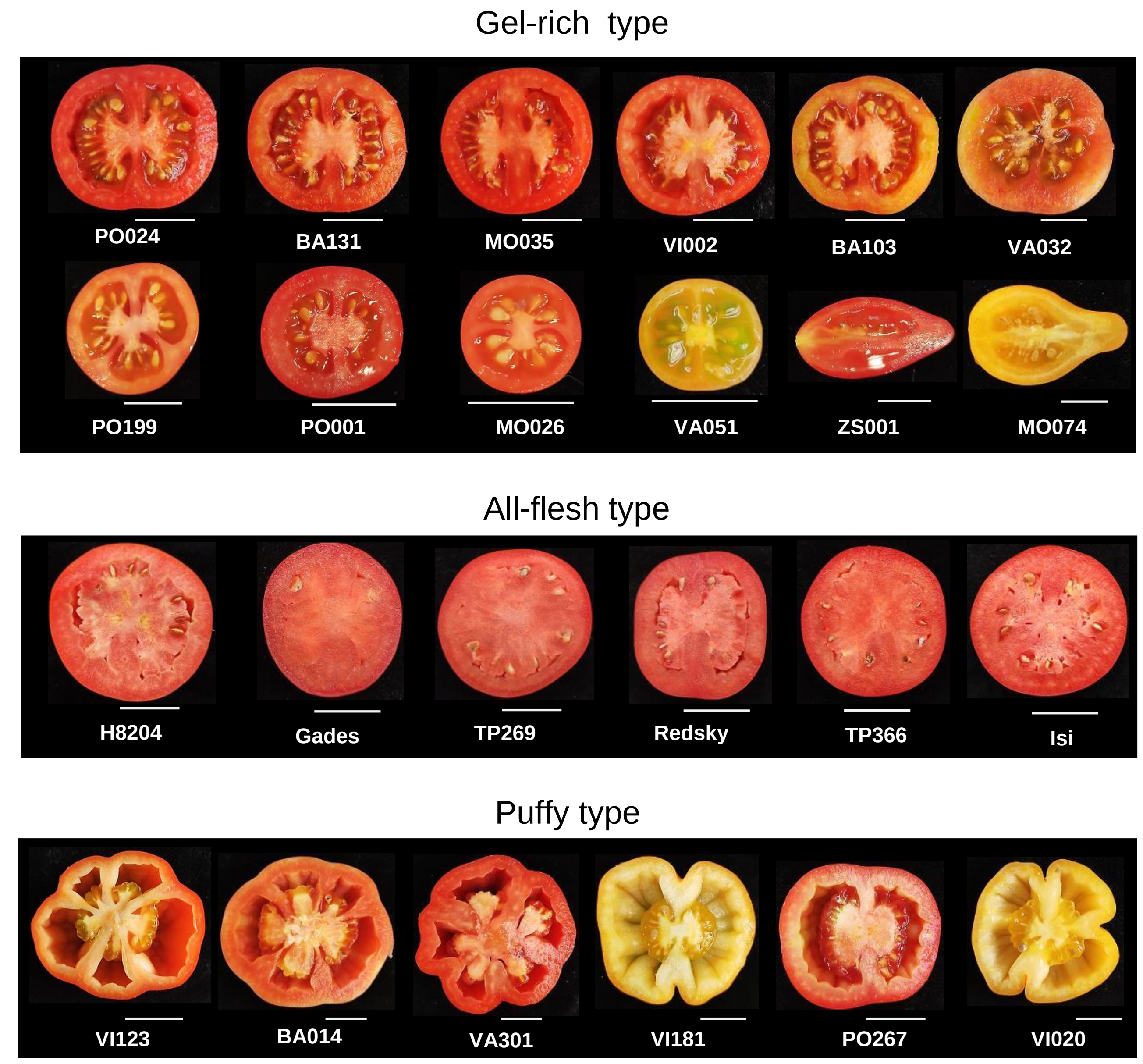

Supplementary Fig. 1. Natural variability of tomato inner tissues structure. The 24 accessions shown illustrate the variability in locular tissue present among tomato commercial cultivars and traditional varieties. Three main types are defined corresponding to Gel-rich with locular cavity filled with liquefied gel, All-flesh with locules filled with a non-jelly tissue, and Puffy with a pepper-like locule. Bars = 2 cm.

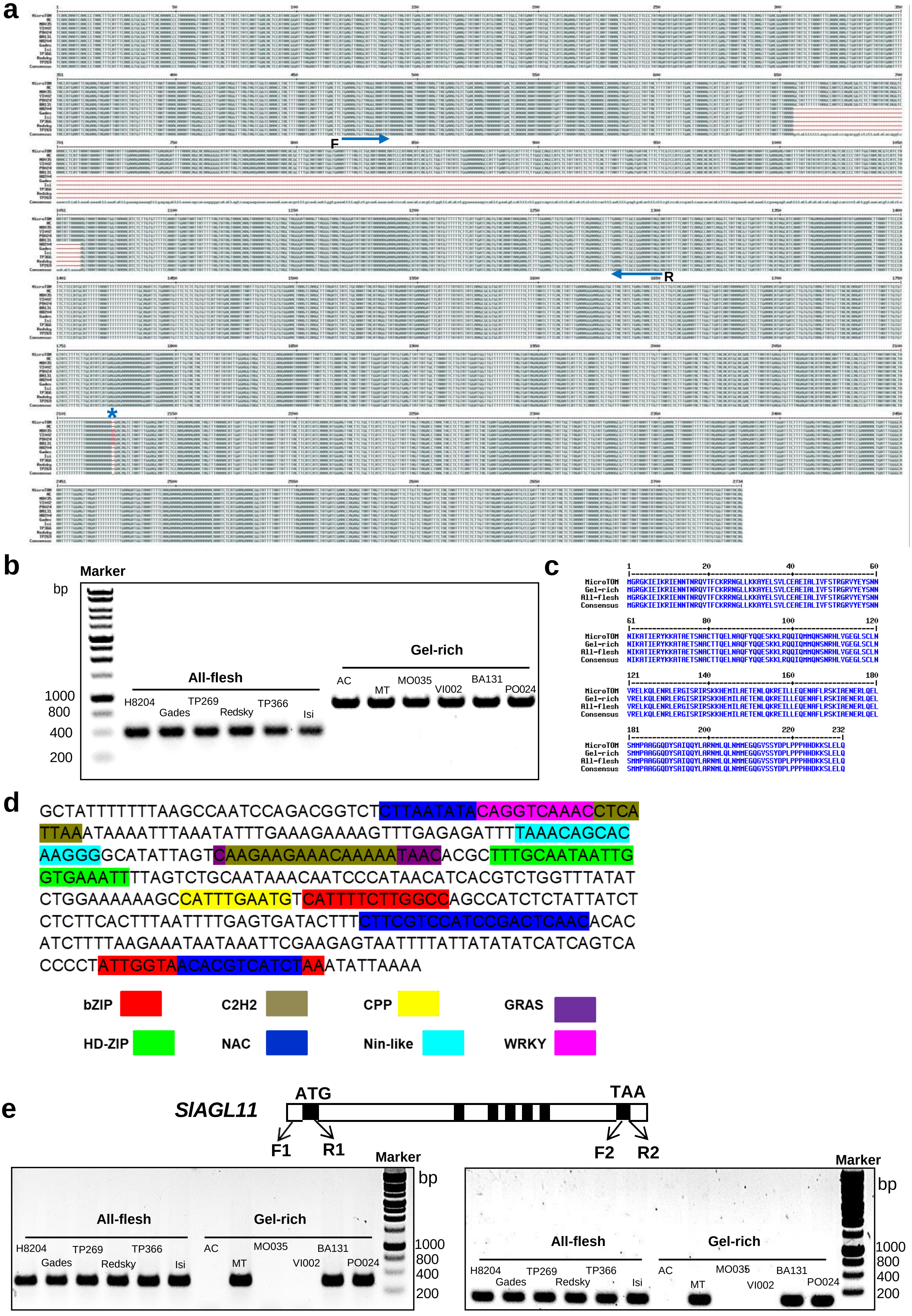

a
F
R
b
Marker
bp
1000
800
400
200
All-flesh
H8204
Gades
TP269
Redsky
TP366
Isi
Gel-rich
AC
MT
MO035
VI002
BA131
PO024
c
MicroTOM
Gel-rich
All-flesh
Consensus
d
GCTATTTTTTTAAGCCAATCCAGACGGTCTCTTAATATACAGGTCAAACCTCA
TTAAATAAAATTTAAATATTTGAAAGAAAAGTTTGAGAGATTTTAAACAGCAC
AAGGGGCATATTAGTCAAGAAGAAACAAAAATAACACGCTTTGCAATAATTG
GTGAAATTTTAGTCTGCAATAAACAATCCCATAACATCACGTCTGGTTTATAT
CTGGAAAAAAGCCATTTGAATGTCATTTTCTTGGCCAGCCATCTCTATTATCT
CTCTTCACTTTAATTTTGAGTGATACTTTCTTCGTCCATCCGACTCAACACAC
ATCTTTTAAGAAATAATAAATTCGAAGAGTAATTTTATTATATATCATCAGTCA
CCCCTATTGGTAACACGTCATCTAAATATTAAAA
bZIP
C2H2
CPP
GRAS
HD-ZIP
NAC
Nin-like
WRKY
e
SlAGL11
ATG
TAA
F1
R1
F2
R2
Marker
bp
All-flesh
Gel-rich
H8204
Gades
TP269
Redsky
TP366
Isi
AC
MT
MO035
VI002
BA131
PO024
1000
800
400
200

Supplementary Fig. 2. Structural analysis of *SlMBP3* and *SlAGL11* loci assessed by PCR and subsequent sequencing. a, Sequence alignment of 2.7kb promoter region of the *SlMBP3* gene in twelve tomato accessions representative of the two locular tissue types. The red line indicates the 405 bp deletion in All-flesh lines and the blue asterisk shows SNP positions. Blue arrows indicate forward and reverse primers (F and R) used in PCR amplification. **b,** Variation within the *SlMBP3* locus revealed by gel electrophoresis of PCR products following amplification on genomic DNA corresponding to the twelve tomato accessions including 6 All-flesh lines and 6 Gel-rich lines. Three independent experiments were performed. **c,** Amino acid sequence alignment of *SlMBP3* CDS from the two types of locular gel. **d,** Analysis of putative Cis-regulatory elements in the 405bp-deleted region of the *SlMBP3* promoter and potential transcription factor binding motifs were highlighted with different colors. **e,** Variation within the *SlAGL11* locus as revealed by gel electrophoresis of PCR amplified fragments within the *SlAGL11* gene of the twelve tomato cultivars. The two amplified fragments are targeted primers F1/R1 and F2/R2 located in exon-1 and exon-7, respectively. Three independent experiments were performed. Marker: 1kb and 100bp DNA ladder.

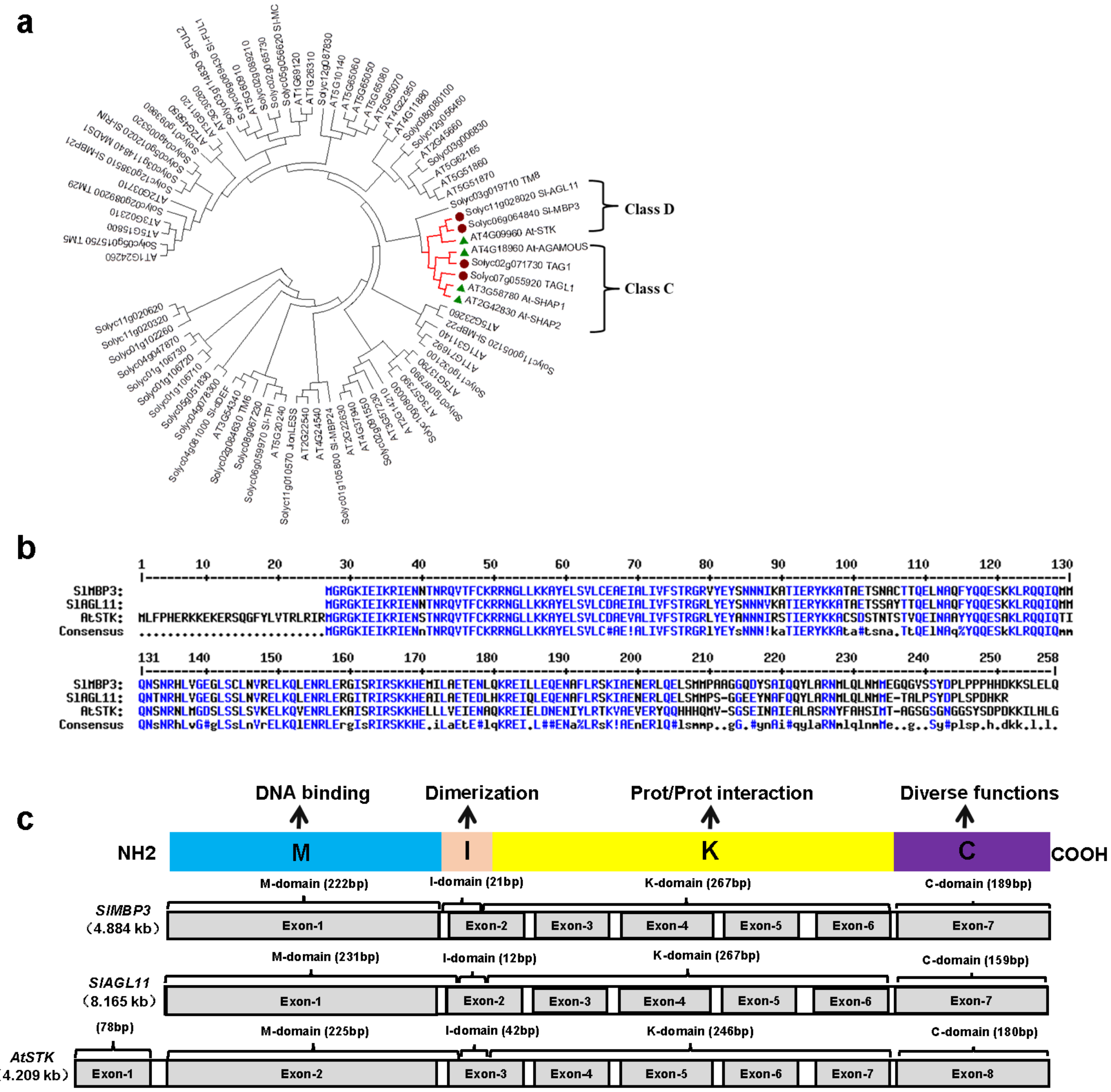

Supplementary Fig. 3. Phylogenetic tree, amino acid sequence and genomic structure of class-D MADS-box genes from Arabidopsis and tomato. a, Phylogenetic tree of MADS-box MIKC type genes in Arabidopsis and tomato obtained by alignment of full-length tomato and Arabidopsis proteins using MUSCLE and clustering with the Maximum Likelihood method. Analyses were conducted in MEGA6. Red circles correspond to tomato genes and green triangles to Arabidopsis thaliana genes. **b,** Amino acid sequence alignment of SlMBP3, SlAGL11 and AtSTK proteins. **c,** Genomic structure and functional domains of SlMBP3, SlAGL11 and AtSTK. Four protein domains: MADS (M-), Intervening(I-), Keratin-like(K-), and C-terminal(C-). Functional domains characteristic of MIKC type plant MADS proteins are well conserved**,** including the M-domain involved in DNA binding, the K-domain involved in protein-protein interactions and dimerization, and the C-domain that has diverse functions. The genomic structure is well conserved between *SlMBP3*, *SlAGL11* and *AtSTK* with the tomato members both comprising 7 exons and the Arabidopsis gene 8 exons. The gene size is 4.884 kb for *SlMBP3*, 8.165 kb for *SlAGL11* and 4.029kb for *AtSTK*.

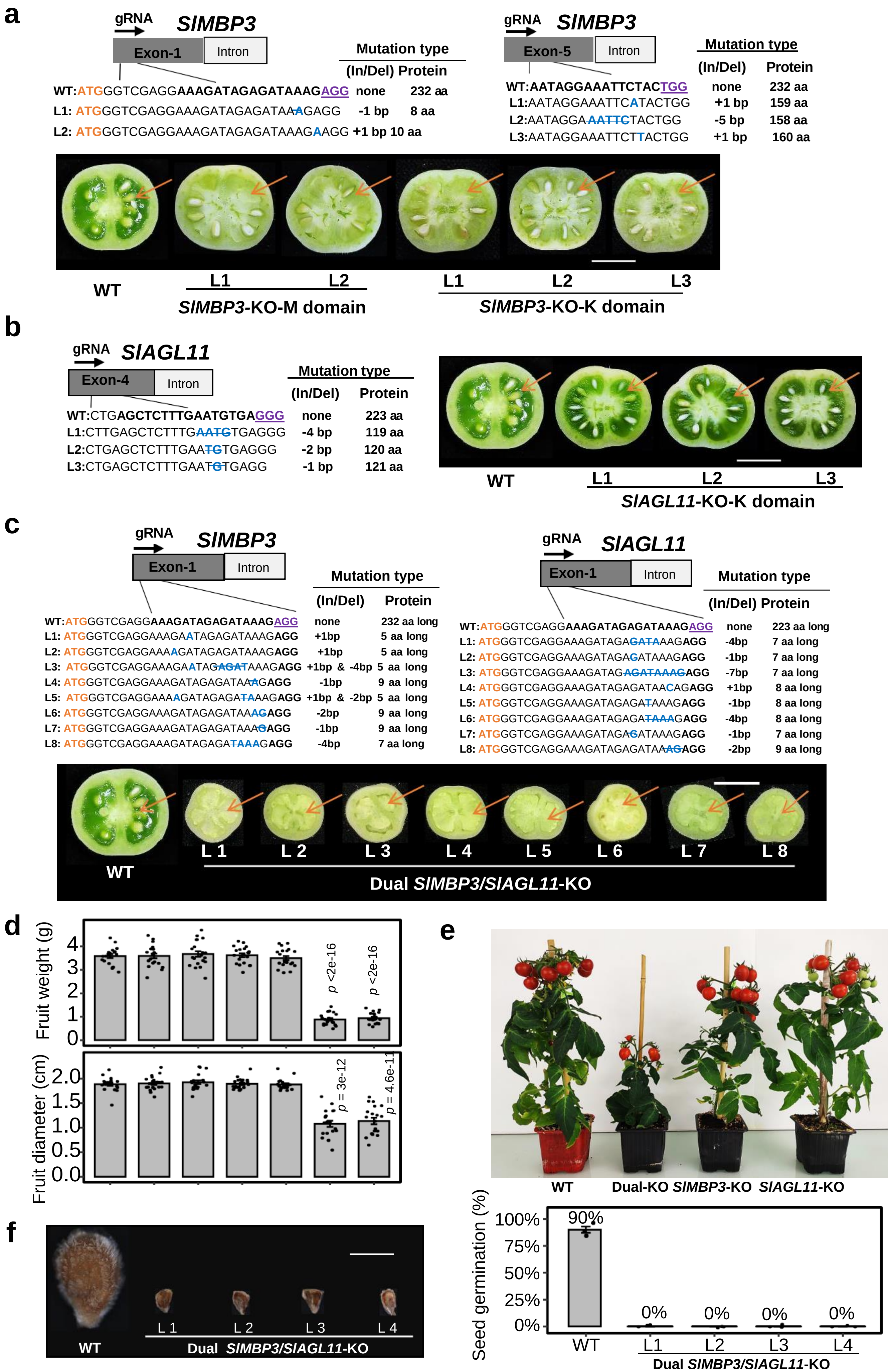

a
gRNA
SlMBP3
Exon-1
Intron
Mutation type
(In/Del) Protein
WT:ATGGGTCGAGGAAAGATAGAGATAAAGAGG none 232 aa
L1: ATGGGTCGAGGAAAGATAGAGATAAAGAGG -1 bp 8 aa
L2: ATGGGTCGAGGAAAGATAGAGATAAAGAAGG +1 bp 10 aa
gRNA
SlMBP3
Exon-5
Intron
Mutation type
(In/Del) Protein
WT:AATAGGAAATTCTACTGG none 232 aa
L1:AATAGGAAATTCATACTGG +1 bp 159 aa
L2:AATAGGAAATTCTACTGG -5 bp 158 aa
L3:AATAGGAAATTCTTACTGG +1 bp 160 aa
WT
L1 L2
SlMBP3-KO-M domain
L1 L2 L3
SlMBP3-KO-K domain
b
gRNA
SlAGL11
Exon-4
Intron
Mutation type
(In/Del) Protein
WT:CTGAGCTCTTTGAATGTGAGGG none 223 aa
L1:CTTGAGCTCTTTGAATGTGAGGG -4 bp 119 aa
L2:CTGAGCTCTTTGAATGTGAGGG -2 bp 120 aa
L3:CTGAGCTCTTTGAATGTGAGG -1 bp 121 aa
WT
L1 L2 L3
SlAGL11-KO-K domain
c
gRNA
SlMBP3
Exon-1
Intron
Mutation type
(In/Del) Protein
WT:ATGGGTCGAGGAAAGATAGAGATAAAGAGG none 232 aa long
L1: ATGGGTCGAGGAAAGAATAGAGATAAAGAGG +1bp 5 aa long
L2: ATGGGTCGAGGAAAAGATAGAGATAAAGAGG +1bp 5 aa long
L3: ATGGGTCGAGGAAAGAATAGAGATAAAGAGG +1bp & -4bp 5 aa long
L4: ATGGGTCGAGGAAAGATAGAGATAAAGAGG -1bp 9 aa long
L5: ATGGGTCGAGGAAAAGATAGAGATAAAGAGG +1bp & -2bp 5 aa long
L6: ATGGGTCGAGGAAAGATAGAGATAAAGAGG -2bp 9 aa long
L7: ATGGGTCGAGGAAAGATAGAGATAAAGAGG -1bp 9 aa long
L8: ATGGGTCGAGGAAAGATAGAGATAAAGAGG -4bp 7 aa long
gRNA
SlAGL11
Exon-1
Intron
Mutation type
(In/Del) Protein
WT:ATGGGTCGAGGAAAGATAGAGATAAAGAGG none 223 aa long
L1: ATGGGTCGAGGAAAGATAGAGATAAAGAGG -4bp 7 aa long
L2: ATGGGTCGAGGAAAGATAGAGATAAAGAGG -1bp 7 aa long
L3: ATGGGTCGAGGAAAGATAGAGATAAAGAGG -7bp 7 aa long
L4: ATGGGTCGAGGAAAGATAGAGATAACAGAGG +1bp 8 aa long
L5: ATGGGTCGAGGAAAGATAGAGATAAAGAGG -1bp 8 aa long
L6: ATGGGTCGAGGAAAGATAGAGATAAAGAGG -4bp 8 aa long
L7: ATGGGTCGAGGAAAGATAGAGATAAAGAGG -1bp 7 aa long
L8: ATGGGTCGAGGAAAGATAGAGATAAAGAGG -2bp 9 aa long
WT
L 1 L 2 L 3 L 4 L 5 L 6 L 7 L 8
Dual SlMBP3/SlAGL11-KO
d
Fruit weight (g)
0 1 2 3 4
p <2e-16
p <2e-16
Fruit diameter (cm)
0.0 0.5 1.0 1.5 2.0
p = 3e-12
p = 4.6e-11
e
WT Dual-KO SlMBP3-KO SlAGL11-KO
Seed germination (%)
0% 25% 50% 75% 100%
90%
0% 0% 0% 0%
WT L1 L2 L3 L4
Dual SlMBP3/SlAGL11-KO
f
WT
L 1 L 2 L 3 L 4
Dual SlMBP3/SlAGL11-KO

Supplementary Fig. 4. *SlMBP3* and *SlAGL11* CRISPR/Cas9-induced mutations and their associated phenotypes. a, Genomic *SlMBP3* sequence showing the gRNA target sites and the fruit phenotype of five independent KO lines as shown by the cross-sections of the fruits at 30-DPA stage. DPA refers to days post-anthesis. Orange arrows point to the locular tissue region with total absence of gel tissue in the KO lines. **b,** Genomic *SlAGL11* sequences showing the gRNA target sites and the fruit phenotype of three independent KO lines as shown by cross sections of the fruits at 30-DPA stage. Orange arrows point to the presence of locular gel in all KO lines. **c,** Genomic *SlMBP3* and *SlAGL11* sequences showing the gRNA target sites and eight independent dual-KO lines all showing the absence of locular gel at 30-DPA stage. The insertion/deletion mutations in both genes leading to truncated proteins lacking the functional domains. Orange arrows point to dual *SlMBP3/SlAGL11*-KO locular tissue area. **d,** Quantification of mean weight and size of different KO lines fruit compared to wild type (WT). For each individual line 20 fruits per stage were used. Values are means ± SD, and significance was determined by two-tailed Student's *t*-test. **e,** Phenotypes of single *SlMBP3*-KO, and *SlAGL11*-KO plants, compared to dual-KO and wild type lines. **f,** The severe seed phenotype in four independent dual *SlMBP3/SlAGL11*-KO lines and their germination rate compared to wild type. Bar = 1 cm for fruit and bar = 1mm for seeds. Source data are provided as a Source Data file.

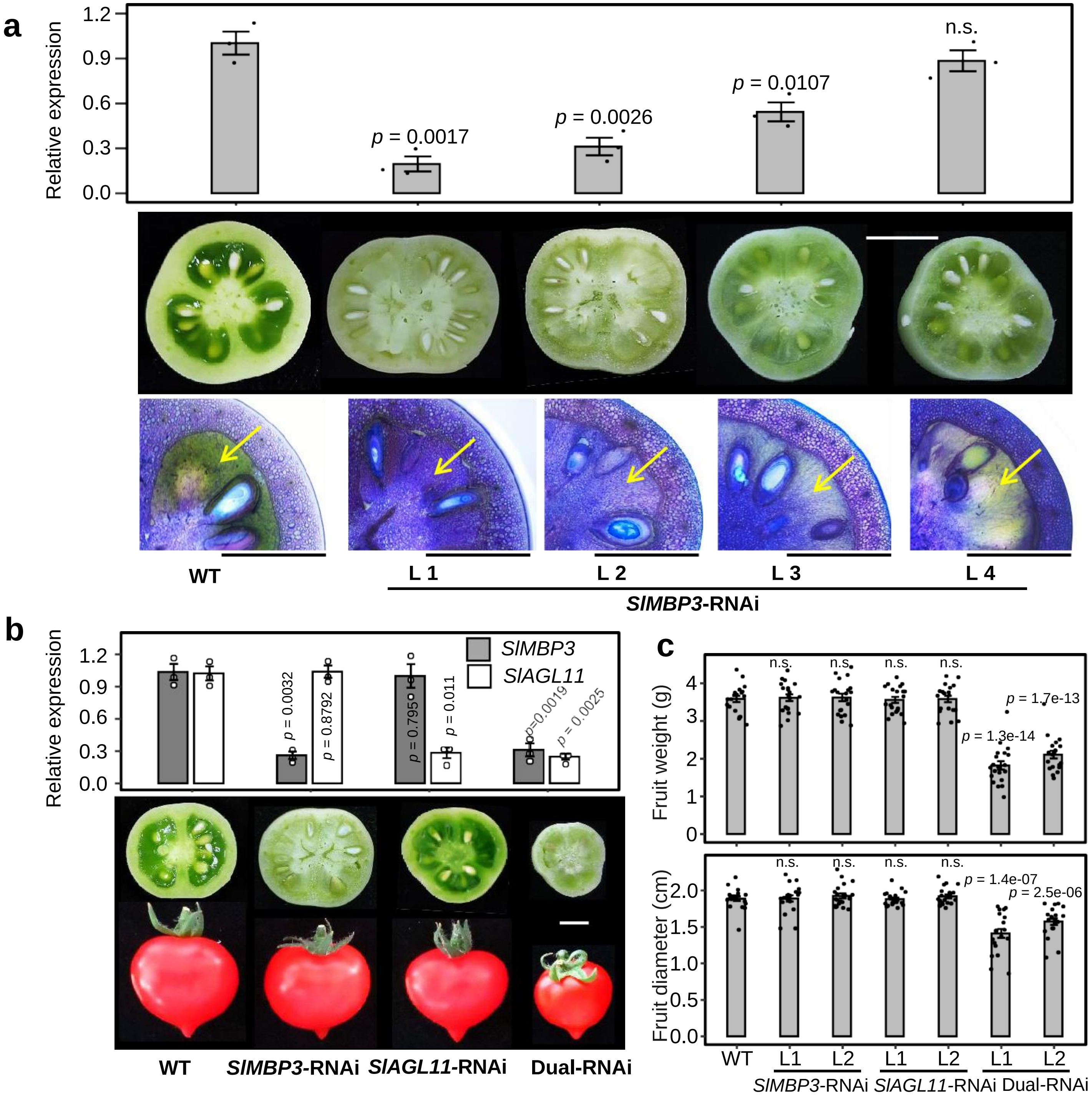

Supplementary Fig. 5. Generation of RNAi down-regulated lines. a, Severity of the phenotypes associated with the level of *SlMBP3* down-regulation in tomato RNAi lines. Four lines exhibiting strong (L1) to mild (L4) down-regulation of *SlMBP3* are presented (upper panel) with the corresponding phenotypes shown (middle panel). Histological observations of fruits sections stained with toluidine blue at 30-DPA stage (lower panel). Yellow arrows point to locular tissue area. Values are means ± standard deviation (SD) of three biological replicates and significance was determined by two-tailed Student's *t*-test. n.s., no significant difference. Bars = 5 mm. **b,** Transcript levels and phenotypes of single and dual *SlMBP3* and *SlAGL11* RNAi fruit. Dual-RNAi correspond to lines expressing RNAi construct targeting a sequence perfectly conserved between *SlMBP3* and *SlAGL11*. Transcript accumulation was assessed by qPCR in 10-DPA fruit tissues. Values are means ± standard deviation (SD) of three biological replicates and significance was determined by two-tailed Student's *t*-test. DPA refers to days post-anthesis. **c,** Mean weight and size of single and dual *SlMBP3* and *SlAGL11* RNAi fruits (n=20). For each individual line 20 fruits per stage were used. Values are means ± SD, and significance was determined by two-tailed Student's *t*-test. n.s., no significant difference. Source data are provided as a Source Data file.

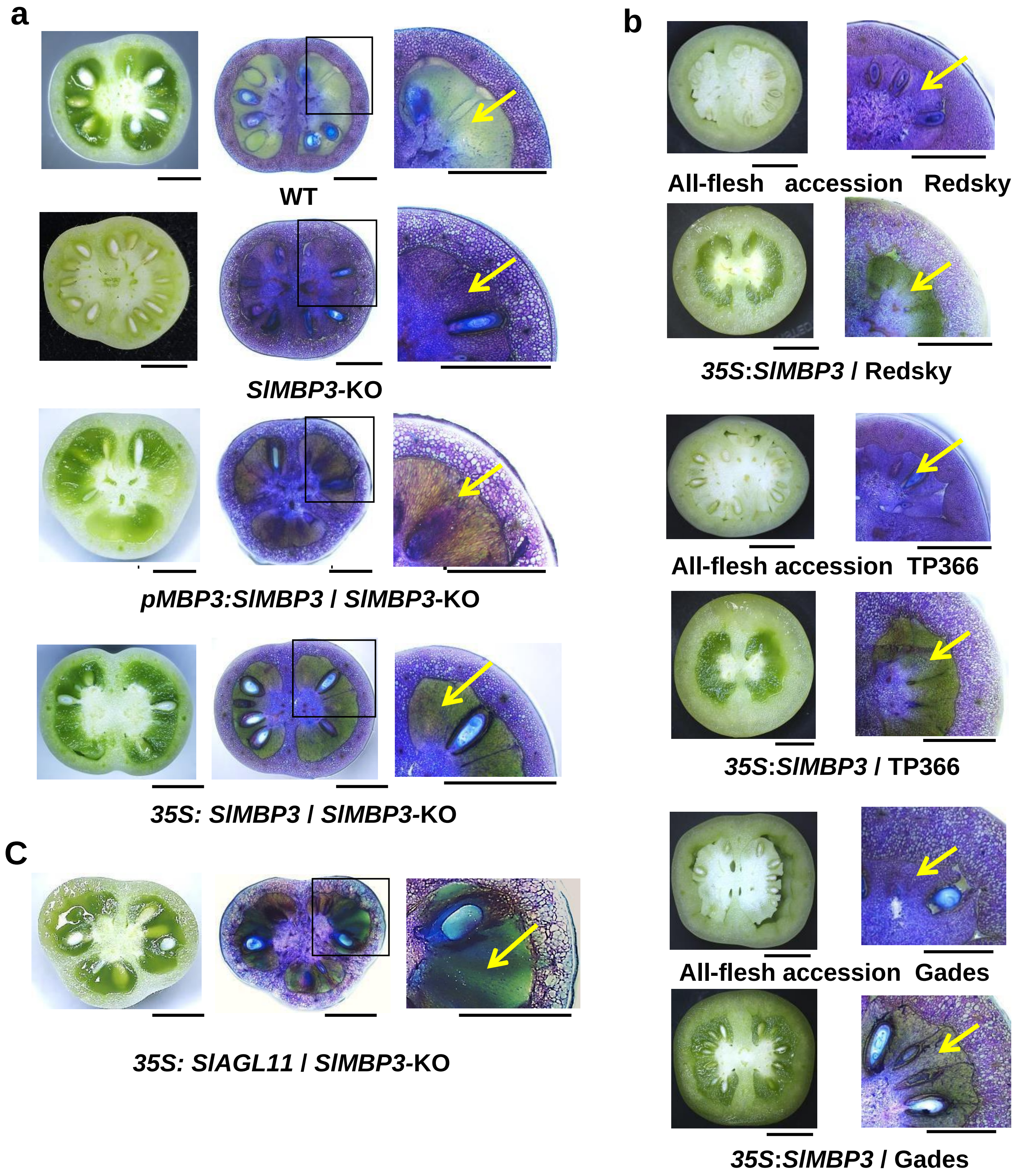

Supplementary Fig. 6. Complementation experiments of the locular gel phenotype in tomato *SlMBP3*-KO mutant lines and All-flesh commercial accessions. a, *SlMBP3*-KO lines were self-complemented via stable genetic transformation with a functional *SlMBP3* gene driven by the native tomato *SlMBP3* gene promoter (*pro*MBP3:*SlMBP3*) or the constitutive 35S promoter (35S:*SlMBP3*). Fruit sections with and without toluidine blue staining are shown for WT (wild type), *SlMBP3*-KO and *SlMBP3*-KO expressing *pro*MBP3:*SlMBP3 or* 35S:*SlMBP3* transgenes. Bar = 5 mm. **b,** The All-flesh tomato accessions Redsky, TP366 and Gades were stably transformed with the 35S::*SlMBP3* transgene leading to a Gel-rich phenotype. All sections were performed with 30-DPA fruit stained with toluidine blue. DPA refers to days post-anthesis. Yellow arrows point to the locular tissue area. The scale bar indicates 1 cm. **c,** *SlMBP3-KO* fruits expressing SlAGL11 driven by the constitutive 35S promoter (*35S:SlAGL11*). Fruit sections with and without toluidine blue staining are shown. Bar = 5 mm.

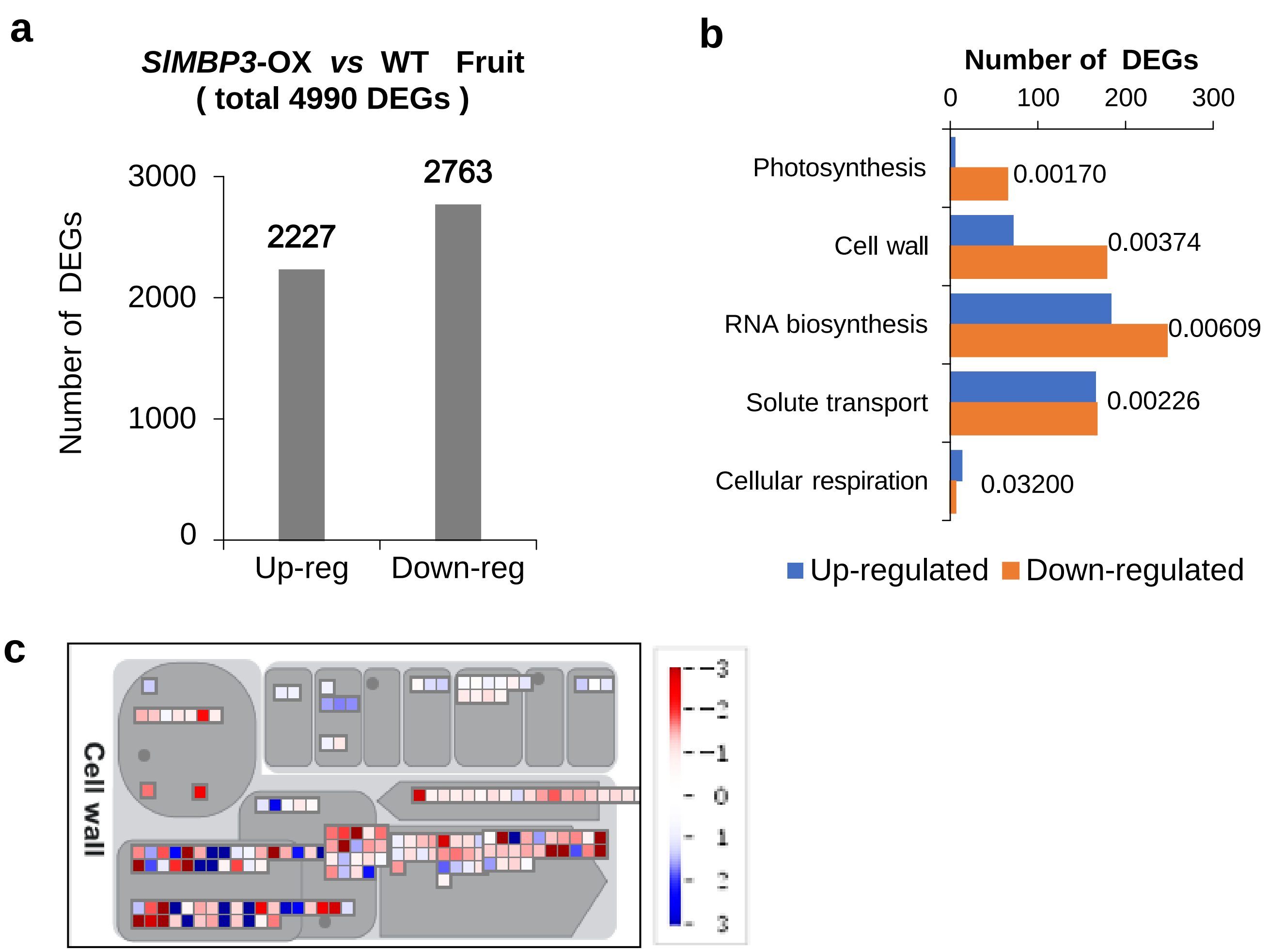

Supplementary Fig. 7. Genome-wide transcriptomic profiling by RNA-seq of *SlMBP3*-OX fruit at 10-DPA stage. a, Differentially expressed genes (DEGs) in *SlMBP3*-OX-fruit. **b,** Top five enriched GO terms in *SlMBP3*-OX fruit as revealed by MAPMAN. Wilcoxon Rank Sum test was used to evaluate the significance (*P*-value<0.05) of term enrichment in each category of candidates. **c,** MAPMAN representation of cell-wall related genes differentially expressed in *SlMBP3*-OX fruit *vs* WT fruit. DEGs were determined using the following rules: basemean>5 and *p-adj*<0.05. DPA refers to days post-anthesis.

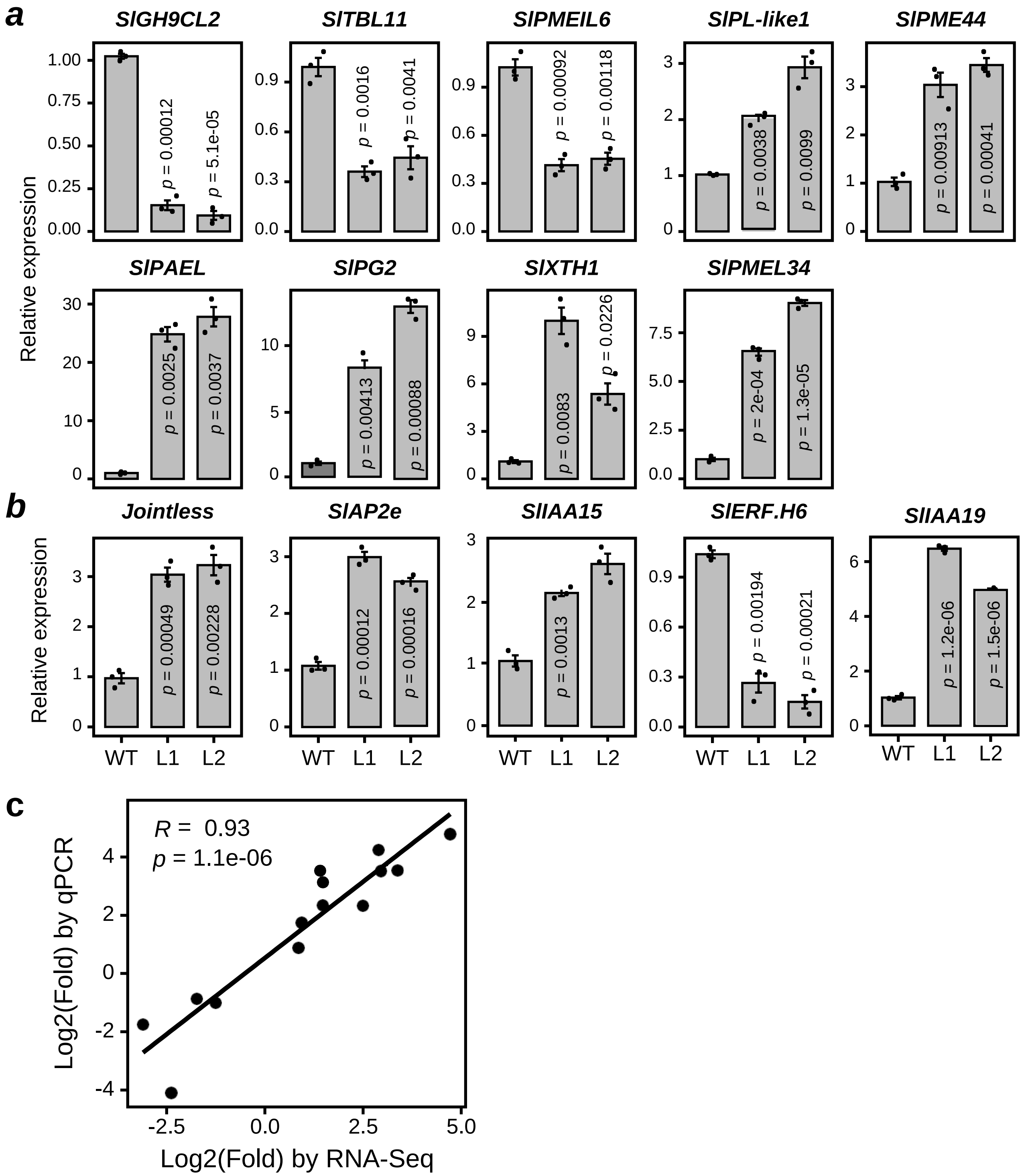

Supplementary Fig. 8. Validation by qPCR of the RNAseq data. a, qPCR analysis of the transcript levels of nine cell wall-related genes identified as differentially expressed by RNA-seq in two independent *SlMBP3*-KO lines. **b,** qPCR analysis in two independent *SlMBP3*-KO lines of the expression level of five transcription factor genes identified as differentially expressed by RNA-seq. The analyses were made with 10-DPA fruit samples. Values are means ± standard deviation (SD) of three biological replicates. Significance was determined by two-tailed Student's *t*-test. **c,** Correlation between RNA-seq and qPCR results are provided for nine cell wall genes and five transcription factors. L1 and L2 are two independent *SlMBP3*-KO lines. The data refer to transcript levels in locular tissue. Source data are provided as a Source Data file.

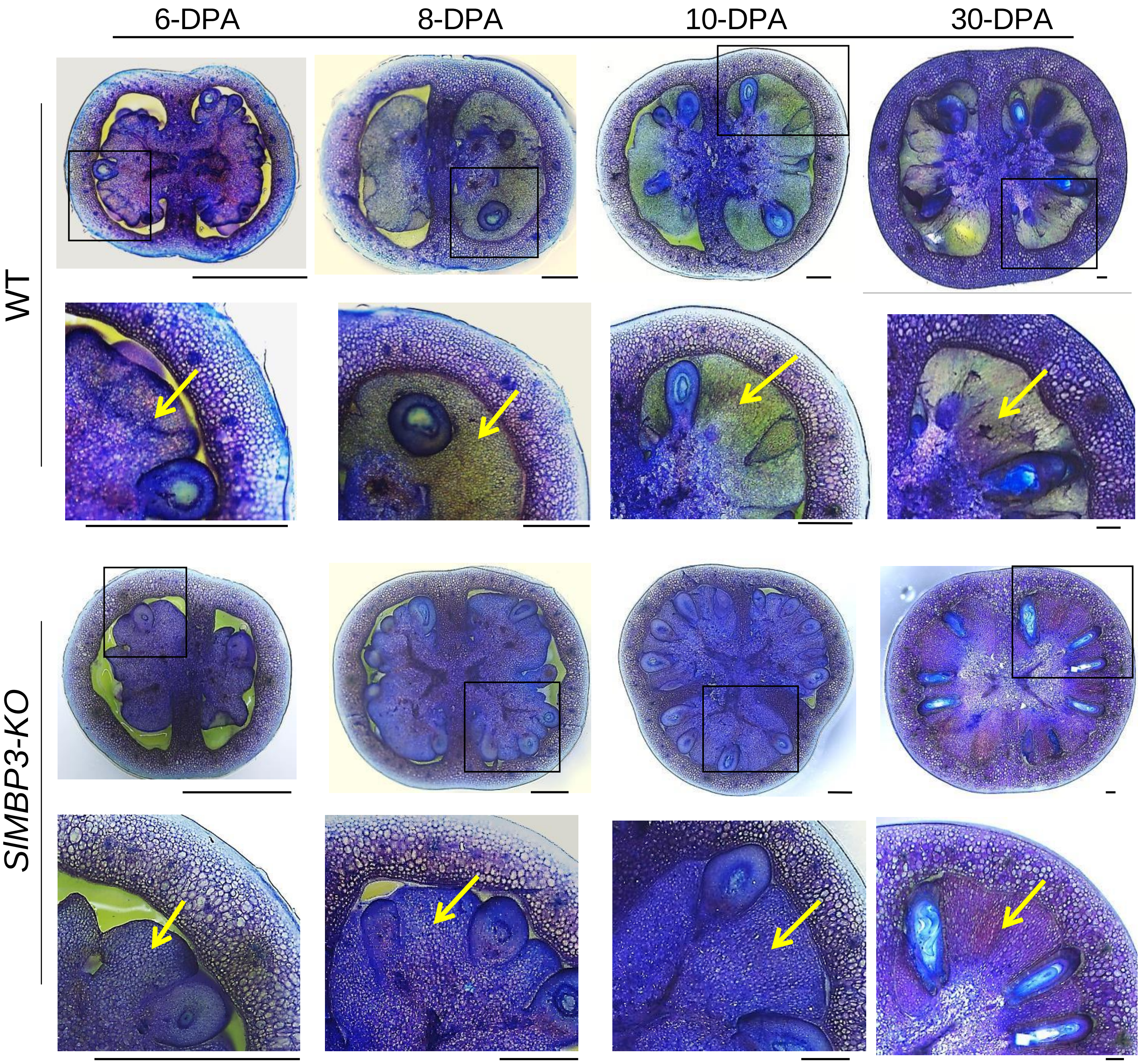

Supplementary Fig. 9. Histological observations of fruit between *SlMBP3*-KO and WT at different development stages. Fruit sections were stained with toluidine blue. DPA refers to days post-anthesis. Scale bar = 1 mm. Yellow arrows point to the locular tissue.

Supplementary Table 1. Cell wall-related genes differentially expressed in *SlMBP3*-KO locular tissue or *SlMBP3*-OX fruit. Differentially expressed genes (DEGs) are defined according to the following rules: basemean>5 and *p-adj*<0.05.

CONTENT	*SlMBP3*-KO-locular tissue		*SlMBP3*-OX-fruit	
	Up-reg	Down-reg	Up-reg	Down-
Cell wall.cutin and suberin.	21	1	1	15
Cell wall.callose synthase	2	0	3	0
Cell wall.cellulose.	19	14	7	20
Cell wall.hemicellulose synthesis	31	12	4	28
Cell wall.lignin	13	3	4	3
Cell wall.modification	10	5	9	10
Cell wall.proteins	30	14	10	25
Cell wall.pectin	56	20	33	59
Cell	2	1	0	0
Cell wall.precursor synthesis	17	4	1	18
Total to cell wall-related DEGs	201	74	72	178
Cell wall-related DEGs / Total	46.7% (275/589)		42.4% (250/589)	
