# Supplementary material for: Interaction of two MADS-box genes leads to growth phenotype divergence of all-flesh type of tomatoes
Source: Nat Commun. 2021 Nov 25;12:6892. doi: 10.1038/s41467-021-27117-7 (PMC8616914; doi:10.1038/s41467-021-27117-7)
Supplement: Supplementary file 3 — Description of Additional Supplementary Files [file 41467_2021_27117_MOESM3_ESM.pdf]

Description of Additional Supplementary Files

File Name: Supplementary Data 1
Description: List of differentially expressed genes (DEGs) in SlMBP3-KO locular tissue vs WT locular gel at 10-DPA stage identified by RNA-seq.

File Name: Supplementary Data 2
Description: List of differentially expressed genes (DEGs) of SlMBP3-OX fruit vs WT fruit at 10-DPA stage by RNA-seq.

File Name: Supplementary Data 3
Description: List of 524 DEGs associated with transcription factor genes in SlMBP3-KO locular tissue at 10-DPA stage.

File Name: Supplementary Data 4
Description: List of total peaks (2363) enriched by ChIP-seq in SlMBP3-GFP fruit at 10-DPA stage.

File Name: Supplementary Data 5
Description: List of 1081 genes defined by ChIP-seq as putative targets of SlMBP3.

File Name: Supplementary Data 6
Description: List of 450 genes defined by ChIP-seq as putative direct targets of SlMBP3 and identified as DEGs by RNA-seq.

File Name: Supplementary Data 7
Description: Cell wall-related DEGs being putative targets of SlMBP3.

File Name: Supplementary Data 8
Description: Transcription factor genes being putative targets of SlMBP3.

File Name: Supplementary Data 9
Description: List of differentially expressed genes (114 GEGs) related to cell cycle and cell division in SlMBP3-KO locular tissue at 10 DPA stage as defined by RNAseq.

File Name: Supplementary Data 10
Description: List of 20 cell cycle and cell division related genes differentially expressed in SlMBP3-KO locular tissue was cross-referenced with putative SlMBP3 targets as defined by ChIP-seq analysis.

File Name: Supplementary Data 11
Description: Primers used in this study.
