# Supplementary material for: Interaction of two MADS-box genes leads to growth phenotype divergence of all-flesh type of tomatoes
Source: Nat Commun. 2021 Nov 25;12:6892. doi: 10.1038/s41467-021-27117-7 (PMC8616914; doi:10.1038/s41467-021-27117-7)
Supplement: Supplementary file 15 — Reporting Summary [file 41467_2021_27117_MOESM15_ESM.pdf]

nature research

Corresponding author(s): Prof.Mondher Bouzayen

Last updated by author(s): Oct 26, 2021

Reporting Summary

Nature Research wishes to improve the reproducibility of the work that we publish. This form provides structure for consistency and transparency in reporting. For further information on Nature Research policies, see our Editorial Policies and the Editorial Policy Checklist.

Statistics

For all statistical analyses, confirm that the following items are present in the figure legend, table legend, main text, or Methods section.

n/a	Confirmed	
☐	☒	The exact sample size (*n*) for each experimental group/condition, given as a discrete number and unit of measurement
☐	☒	A statement on whether measurements were taken from distinct samples or whether the same sample was measured repeatedly
☐	☒	The statistical test(s) used AND whether they are one- or two-sided<br>*Only common tests should be described solely by name; describe more complex techniques in the Methods section.*
☒	☐	A description of all covariates tested
☒	☐	A description of any assumptions or corrections, such as tests of normality and adjustment for multiple comparisons
☐	☒	A full description of the statistical parameters including central tendency (e.g. means) or other basic estimates (e.g. regression coefficient) AND variation (e.g. standard deviation) or associated estimates of uncertainty (e.g. confidence intervals)
☐	☒	For null hypothesis testing, the test statistic (e.g. *F*, *t*, *r*) with confidence intervals, effect sizes, degrees of freedom and *P* value noted<br>*Give P values as exact values whenever suitable.*
☒	☐	For Bayesian analysis, information on the choice of priors and Markov chain Monte Carlo settings
☒	☐	For hierarchical and complex designs, identification of the appropriate level for tests and full reporting of outcomes
☐	☒	Estimates of effect sizes (e.g. Cohen's *d*, Pearson's *r*), indicating how they were calculated

Our web collection on statistics for biologists contains articles on many of the points above.

Software and code

Policy information about availability of computer code

Data collection	ZEN microscope software (Zeiss, Germany) (FR-AIB TRI imaging platform); QS6 sequence detection system (Applied Biosystems, USA)
Data analysis	TrimGalore (Version 0.6.5); cutadapt (Version 1.7.1); FastQC (Version 0.11.9);BWA (Burrows-Wheeler Aligner, version 0.7.17); GATK (version 4.1.9.0); HaplotypeCaller (version 4); Integrative Genomic Viewer (IGV2.0);MEGA6;HISAT2 2.1.0;DESeq2 R package;MAPMAN software (version 3.5.1); MACS2 v1.4.2; MEME-ChIP; ComplexHeatmap R package; NDPview; ImageJ Version 1.50i; SAMtools 1.3.1; R (version 4.0.0)

For manuscripts utilizing custom algorithms or software that are central to the research but not yet described in published literature, software must be made available to editors and reviewers. We strongly encourage code deposition in a community repository (e.g. GitHub). See the Nature Research guidelines for submitting code & software for further information.

Data

Policy information about availability of data

All manuscripts must include a data availability statement. This statement should provide the following information, where applicable:

- Accession codes, unique identifiers, or web links for publicly available datasets
- A list of figures that have associated raw data
- A description of any restrictions on data availability

All data supporting the findings of this study are available within the article and its supplementary information files; All high-throughput sequencing data have been deposited in the European Nucleotide Archive (http://www.ebi.ac.uk/ena/data/) (accession number: PRJEB43158). Source data are provided with this paper.

Field-specific reporting

Please select the one below that is the best fit for your research. If you are not sure, read the appropriate sections before making your selection.

☒ Life sciences ☐ Behavioural & social sciences ☐ Ecological, evolutionary & environmental sciences

For a reference copy of the document with all sections, see nature.com/documents/nr-reporting-summary-flat.pdf

Life sciences study design

All studies must disclose on these points even when the disclosure is negative.

Sample size	For fruit firmness , fruit weight and other assays, more than then fruits were used to reduce measurement error.
Data exclusions	No data were excluded in this study.
Replication	All presented in figure legends and methods. Individual replicates are indicated and at least 3 independent replicates analyzed for each experiment.
Randomization	Samples were grown on the same condition and randomly allocated in the growth chamber. Experimental plant materials were collected randomly without any bias.
Blinding	No blinding. The experimental materials are plants, thus the blinding design is not applicable to this system.

Reporting for specific materials, systems and methods

We require information from authors about some types of materials, experimental systems and methods used in many studies. Here, indicate whether each material, system or method listed is relevant to your study. If you are not sure if a list item applies to your research, read the appropriate section before selecting a response.

Materials & experimental systems

n/a	Involved in the study	
☐	☒	Antibodies
☒	☐	Eukaryotic cell lines
☒	☐	Palaeontology and archaeology
☒	☐	Animals and other organisms
☒	☐	Human research participants
☒	☐	Clinical data
☒	☐	Dual use research of concern

Methods

n/a	Involved in the study	
☐	☒	ChIP-seq
☒	☐	Flow cytometry
☒	☐	MRI-based neuroimaging

Antibodies

Antibodies used	For ChIP, following two antibodies were used:GFP antibodies (Abcam,AB290) and IgG antibodies (Emd Millipore, 12370).
Validation	The validation statements for all the commercial antibodies used in this study can be found in the manufacturers' websites through catalogue numbers. These antibodies were used for ChIP in many studies, for example, Qi H et al.,2020 plant cell; Zhuang H et al.,2020 plant cell; Liu F et al.,2020 plant cell; Zhang B et al.,2020 Nature.

ChIP-seq

Data deposition

☒ Confirm that both raw and final processed data have been deposited in a public database such as [GEO](GEO).

☒ Confirm that you have deposited or provided access to graph files (e.g. BED files) for the called peaks.

Data access links<br>*May remain private before publication.*	All high-throughput sequencing data have been deposited in the European Nucleotide Archive (http://www.ebi.ac.uk/ena/data/) (accession number: PRJEB43158).
Files in database submission	MBP3GFP_R1.fastq.gz<br>MBP3GFP_R2.fastq.gz<br>MBP3Input_R1.fastq.gz<br>MBP3Input_R2.fastq.gz

Genome browser session (e.g. UCSC)	no longer applicable

Methodology

Replicates	ChIP: 2 replicates (a pool of DNA from 6 plants per replicate) were performed in this study.
Sequencing depth	Total number of paired-end reads for each sample: >3 million reads;<br>Length of reads: 150 bp;
Antibodies	GFP antibodies (Abcam,AB290) and IgG antibodies (Emd Millipore, 12370)
Peak calling parameters	macs2 callpeak -t MBP3_dedupliate.bam -c input_dedupliate.bam -f BAM -g 7.78e8 -s 147 --bw=234 -B
Data quality	Reads were eliminated when the average quality score of all the bases was lower than 25 using Trimmomatic-0.36. Thus, average reliability of bases is more than 99.6%.
Software	Trimmomatic-0.36, Bowtie2 2.2.9, SAMtools 1.3.1, BEDTools 2.17.0, IGV 2.3.25 and MACS2 v1.4.2 were used for ChIP-seq data processing.
